# Mortality from oral and oropharyngeal cancer: age-period-cohort effect, Brazil, 1983–2017

**DOI:** 10.11606/s1518-8787.2021055003093

**Published:** 2021-10-25

**Authors:** Lillia Magali Estrada Perea, José Leopoldo Ferreira Antunes, Marco Aurelio Peres

**Affiliations:** I Universidade Federal de Santa Catarina Programa de Pós-graduação em Saúde Coletiva FlorianópolisSC Brasil Universidade Federal de Santa Catarina. Programa de Pós-graduação em Saúde Coletiva. Florianópolis, SC, Brasil; II Universidade de São Paulo Faculdade de Saúde Pública São PauloSP Brasil Universidade de São Paulo. Faculdade de Saúde Pública. São Paulo, SP, Brasil; III National Dental Research Institute Singapore National Dental Centre Singapore Singapore National Dental Research Institute Singapore. National Dental Centre Singapore. Singapore

**Keywords:** Oropharyngeal Neoplasms, Mortality, Age Effect, Period Effect, Cohort Effect

## Abstract

**OBJECTIVE:**

Estimate the effect of age, period, and birth cohort on mortality from oral and oropharyngeal cancer in Brazil and its macro-regions.

**METHODS:**

Deaths from oral and oropharyngeal cancer from 1983 to 2017 were analyzed. The Poisson regression model was applied, using estimable functions proposed by Holford.

**RESULTS:**

From 1983 to 2017, 142,634 deaths from oral and oropharyngeal cancer were registered in Brazil, 81% among men, and the South and Southeast regions had the highest rates. The most significant period effects were observed in male mortality in the Southeast and Central-West regions for the 2003–2007 reference period. In the North, Northeast, and Central-West regions, an increased risk of mortality was observed in the most recent male cohorts. In the North region, the most significant risk identified was for men born during 1973–1977 (RR = 1.47; 95%CI 1.05–2.08); in the Northeast, for men born during 1988–1992 (RR = 2.77; 95%CI 1.66–4.63); and in the Central-West, for women born during 1973–1977 (RR = 2.01; 95%CI 1.19–3.39). In the Southeast and South regions, the most recent cohorts had lower mortality rates. The lowest risk in the Southeast region was observed in the male cohort born during 1978–1982 (RR = 0.53; 95%CI 0.45–0.62) and 1983–1987 in the South region (RR = 0.25; 95%CI 0.12–0.54).

**CONCLUSIONS:**

Age had a significant effect on mortality from oral and oropharyngeal cancer in all regions. In the North, Northeast, and Central-West regions, an increase in risk was observed in the most recent cohorts, while in the South and Southeast regions, these cohorts presented a lower risk when compared to the older cohorts.

## INTRODUCTION

As a result of the population aging that characterizes the demographic transition, increasing the incidence of chronic non-communicable diseases (NCDs), the Brazilian morbidity and mortality profile has changed^1^ . Within the group of NCDs, in 2017, cancer was responsible for 56.9% of deaths in Brazil in the age group 30–69 years^2^ . The growth in healthcare spending on cancer observed in recent decades is precisely due to the age composition of the population^1^ .

Oral and oropharyngeal cancer is considered a public health problem, especially in Brazil, the country with the highest mortality rates from this type of cancer in Latin America^3^ . Increased incidence of this neoplasm and an increasing trend from 1983 to 2002 have been observed predominantly in high-income countries^4^ .

Despite progress in research and therapy, the survival of between 5 and 10 years of patients diagnosed with oral and oropharyngeal cancer has not significantly improved in recent years^5^ . In 2018, oral cancer had the highest incidence of all cancers in Melanesia and South Asia among men. It was the leading cause of cancer-related mortality among men in India and Sri Lanka. In countries with a low Human Development Index, mouth cancer is the fourth most common type of cancer among men^6 , 7^ .

The analysis of indicators over time is helpful as it allows detecting factors that affect population groups differently. Dealing with data or observations ordered over time requires analyzing and interpreting the contributions of three key phenomena: age effect, period effects, and effects of differences in the year of birth also called “cohort effects”, through a systematic study called “age-period-cohort analysis” (APC)^8^ .

Studies on the historical behavior of mortality from oral and pharyngeal cancer are generally limited to analyzing the historical series of standardized rates, valuable indicators to measure the effect of age and period. However, this analysis leaves out the possible effect of the birth cohort on the behavior of chronic diseases.

This study aims to estimate for the first time the effects of age, period, and birth cohort on mortality from oral and oropharyngeal cancer in macro-regions of Brazil.

## METHODS

This is an ecological study of the temporal distribution of mortality from oral and oropharyngeal cancer in Brazil and its macro-regions from 1983 to 2017, using the APC model. The study considered the deaths of people aged 25 years and over since oral and oropharyngeal cancer cases in the population under 25 years of age are rare (< 2% of all cases). The first cohort analyzed included those born between 1903 and 1907.

Mortality data were obtained from the Datasus Mortality Information System (SIM)^9^ . Deaths from oral and oropharyngeal cancer were included (140, 141, 143-146, 149 from ICD 9^th^ Revision, e C00-C06, C09, C10, C14 from ICD 10^th^ Revision) according to the table of correspondence proposed by Fritz et al^10^ . Population data were also obtained from Datasus, based on 1980, 1991, 2000, and 2010 censuses. The projections for populations in the inter-census years were estimated by the Brazilian Institute of Geography and Statistics (IBGE)^11^ . SIM data are cataloged as secondary data without identifying the patients’ names and, therefore, do not imply a risk for subjects, information, or families.

Studies using secondary data may be affected by underreporting in recording information. To minimize this problem, deaths from ill-defined causes (ICD-9 codes 780-799 and ICD-10 R00-R99) were redistributed proportionally to oral and oropharyngeal cancer cases each year sex, and age group^12^ . The age groups used were grouped into 5-year intervals.

For analysis of the APC model, the periods were grouped into five-year intervals, totaling seven periods, and the Poisson regression model was used. This model assumes that the expected number of deaths follows a Poisson distribution and can be expressed as a log-linear regression, as observed in equation [1]:

$$log\;(E_{ij})\;=\;log\;(P_{ij})\;+\;\mu \;+\;\alpha_{i\;+}\;\beta_{j\;+}\;{ϓ}_{k\;+}\;\varepsilon_{ij}$$ [1]

Where ( *E*_ij_) denotes the expected number of deaths in the group ( *i* , *j* ), and the log of ( *P*_ij_) is the exposure or time each individual was exposed to risk, also called the offset; *µ* represents the intercept; α_i_ represents the effect of age group *i* ; β_j_ represents the effect of period *j* ; ϓ_k_ is the cohort effect *k* . The term ε_ij_ is relative to the random error for age *i* and period *j* ^13^ .

The main difficulty in adjusting a model involving age, period, and cohort is the linear relationship between them, which sets up a problem known as the “problem of non-identifiability.” There is no consensus on the best way to resolve it. The present study chose to estimate the parameters of the APC effect using deviations, curvatures, and drift, a method proposed by Holford^14^ , widely used and accepted in the literature on cancer mortality. This method suggests limiting the analysis of effects to their linear combinations and curvatures. The linear trend of the effects is divided into a first linear effect associated with age and a second effect called drift, the linear effect of period and cohort.

The association generated by the APC model is the relative risk (RR) of each period for 2003–2007 and each cohort’s RR for the cohort of those born during 1943–1947. These references were chosen considering that the cohorts and central periods have greater stability^14^ . The deviance statistic was used to assess the fit of the model. The contribution of the effects was evaluated by comparing the deviance of the estimated model with the specific effect concerning the complete model (age-period-cohort). Statistically significant values were determined by analyzing 95% confidence intervals. Analyses were performed with the Epi library of the free R software (R Foundation for Statistical Computing, Vienna, Austria).

## RESULTS

From 1983 to 2017, 142,634 deaths from oral and oropharyngeal cancer were registered in Brazil, 139,924 (98.1%) among people aged 25 years or more. Among these deaths of individuals older than 25 years, 81% occurred among men. Mortality rates for men were, on average, five times higher than for women. The Southeast region presented rates twice as high as the region with the lowest rates, the Northeast region.

The Southeast and South regions had the highest average rates for the period, 6.4 per 100,000 inhabitants and 6.2 per 100,000 male inhabitants, and 1.1 per 100,000 inhabitants and 1 per 100,000 female inhabitants. The lowest average rates were observed in the North region, 2.4 per 100,000 male inhabitants and 0.9 per 100,000 female inhabitants.

As shown in Table 1 , the APC model showed the best fit compared to the other models (age, age-drift, age-cohort, age-period).

**Table 1:** Adjustments of the APC effect models for mortality from oral and oropharyngeal cancer between 1983 and 2017 in Brazil, according to sex and regions.

North						

Model	Men			Women		
	
	Degrees of freedom	Deviance	Model	Degrees of freedom	Deviance	Model
Age	72	136.56		72	109.97	
Age-drift	71	95.43	< 0.001	71	109.97	0.994
Age-cohort	55	74.57	0.184	55	90.27	0.234
**Age-period-cohort**	**50**	**59.67**	**0.010**	**50**	**75.24**	**0.010**
Age-period	66	81.29	0.156	66	97.10	0.147
Age-drift	71	95.43	0.014	71	109.97	0.024

**Northeast**						

**Model**	**Men**			**Women**		
	
	**Degrees of freedom**	**Deviance**	**Model**	**Degrees of freedom**	**Deviance**	**Model**

Age	72	1408.09		72	359.20	
Age-drift	71	265.50	< 0.001	71	180.35	< 0.001
Age-cohort	55	171.60	< 0.001	55	125.04	< 0.001
**Age-period-cohort**	**50**	**64.02**	**< 0.001**	**50**	**67.65**	**< 0.001**
Age-period	66	118.25	< 0.001	66	97.01	0.021
Age-drift	71	265.50	< 0.001	71	180.35	< 0.001

**Southeast**						

**Model**	**Men**			**Women**		
	
	**Degrees of freedom**	**Deviance**	**Model**	**Degrees of freedom**	**Deviance**	**Model**

Age	72	1105.14		72	204.61	
Age-drift	71	847.40	< 0.001	71	135.36	< 0.001
Age-cohort	55	380.05	< 0.001	55	77.68	< 0.001
**Age-period-cohort**	**50**	**78.11**	**< 0.001**	**50**	**47.25**	**< 0.001**
Age-period	66	397.09	< 0.001	66	91.98	< 0.001
Age-drift	71	847.40	< 0.001	71	135.36	< 0.001

**South**						

**Model**	**Men**			**Women**		
	
	**Degrees of freedom**	**Deviance**	**Model**	**Degrees of freedom**	**Deviance**	**Model**

Age	72	319.87		72	84.11	
Age-drift	71	274.06	< 0.001	71	70.21	< 0.001
Age-cohort	55	193.57	< 0.001	55	55.12	0.518
**Age-period-cohort**	**50**	**64.10**	**< 0.001**	**50**	**42.33**	**0.025**
Age-period	66	123.85	< 0.001	66	56.60	0.578
Age-drift	71	274.06	< 0.001	71	70.21	0.018

**Central-West**						

**Model**	**Men**			**Women**		
	
	**Degrees of freedom**	**Deviance**	**Model**	**Degrees of freedom**	**Deviance**	**Model**

Age	72	273.92		72	120.48	
Age-drift	71	161.83	< 0.001	71	116.45	0.044
Age-cohort	55	117.21	< 0.001	55	99.98	0.420
**Age-period-cohort**	**50**	**55.05**	**< 0.001**	**50**	**66.85**	**< 0.001**
Age-period	66	105.46	< 0.001	66	84.37	0.352
Age-drift	71	161.83	< 0.001	71	116.45	< 0.001

**Figure 1:**
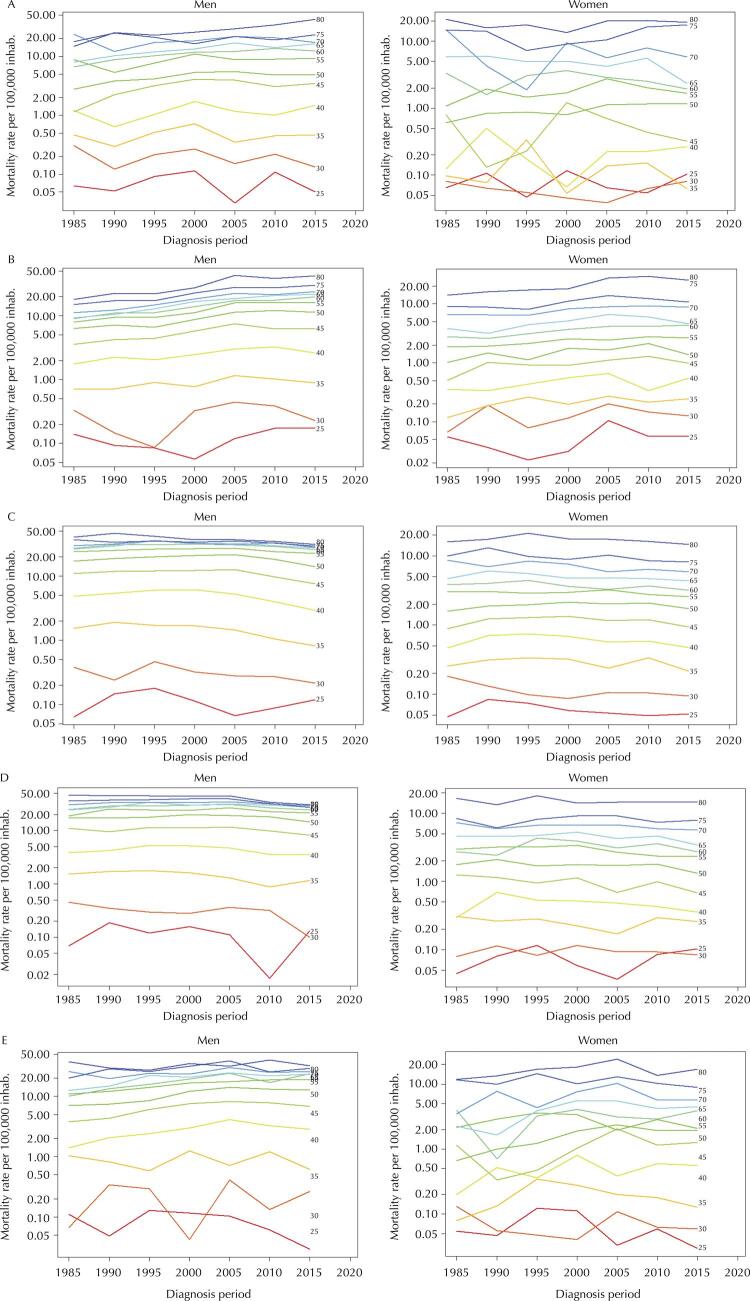
Mortality rates for oral and oropharyngeal cancer by period, connected within each age group, according to sex and region. Brazil, 1983–2017.

Figure 1 presents the behavior of mortality rates within each age group in the different periods analyzed. Age effect on mortality from oral and oropharyngeal cancer is evident in all regions since older age groups always present the highest rates. The parallelism observed between the lines of the different age groups shows the absence of a substantial period effect, which was also observed in the effects obtained by the APC model. The lower lines, corresponding to the mortality rates for the younger age groups, indicate the instability of the rates due to the low number of cases in these groups.

**Figure 2:**
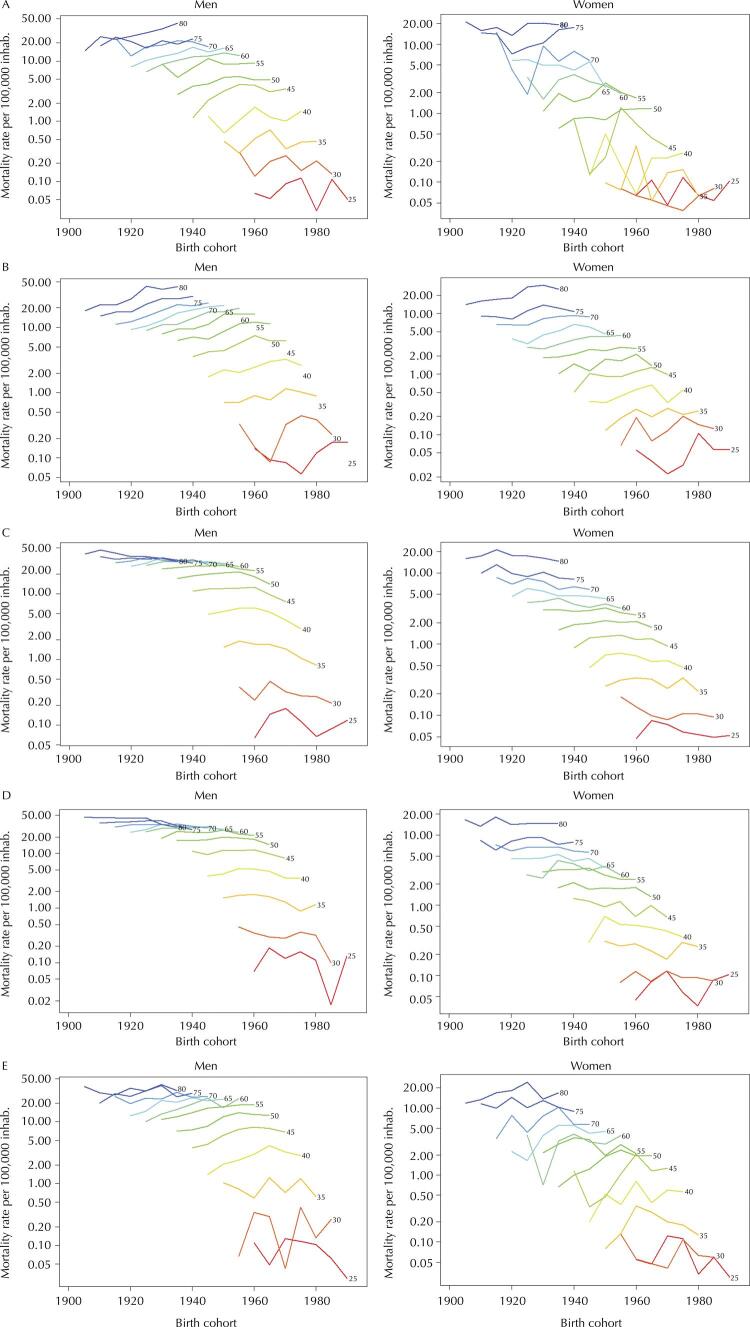
Mortality rates for oral and oropharyngeal cancer by cohort, connected within each age group, by sex and region. Brazil, 1983–2017.

Figure 2 , on the other hand, shows the mortality rates within each age group, but this time according to the analyzed cohorts. The North ( Figure 2A ), Northeast ( Figure 2B ), and Central-West ( Figure 2E ) regions present a positive slope of the lines corresponding to each age group, which indicates an increase in rates for the younger cohorts. Conversely, this trend is negative in the Southeast ( Figure 2C ) and South ( Figure 2D ) regions. This result, regarding the cohorts, was also found in the effects obtained by the APC model. Again, the lower lines show rate instability due to the small number of cases in these groups.

The results obtained by the APC model showed that age is the effect that most influences mortality rates for oral and oropharyngeal cancer. There was a significant increase in mortality in the North, Northeast, and Central-West regions from older ages (45 years for men and 60 for women). The increase in mortality does not behave linearly across all age groups and the slope changes in older age groups. In the Southeast and South regions, the increased risk of death is already present in younger age groups, from 35 years old for men and 45 years old for women. When analyzing the country as a whole, there is significantly increased mortality from 40 years of age for men and 50 years for women.

No significant period effects were observed for the North and Southeast regions. In the Northeast, all periods were at lower risk when compared to the reference, and the period of least risk was 1993–1997 for men (RR = 0.80, 95%CI 0.76–0.84) and 2013–2017 for women (RR = 0.78, 95%CI 0.72–0.83). In the South, the period effect was only observed among men, who during the periods 1983–1987 and 1988–1993 had a lower risk compared to the reference (RR = 0.81, 95%CI 0.78–0.85; and RR = 0.89, 95%CI 0.86–0.94, respectively). In the Central-West, the period of most significant risk was the reference period.

**Figure 3:**
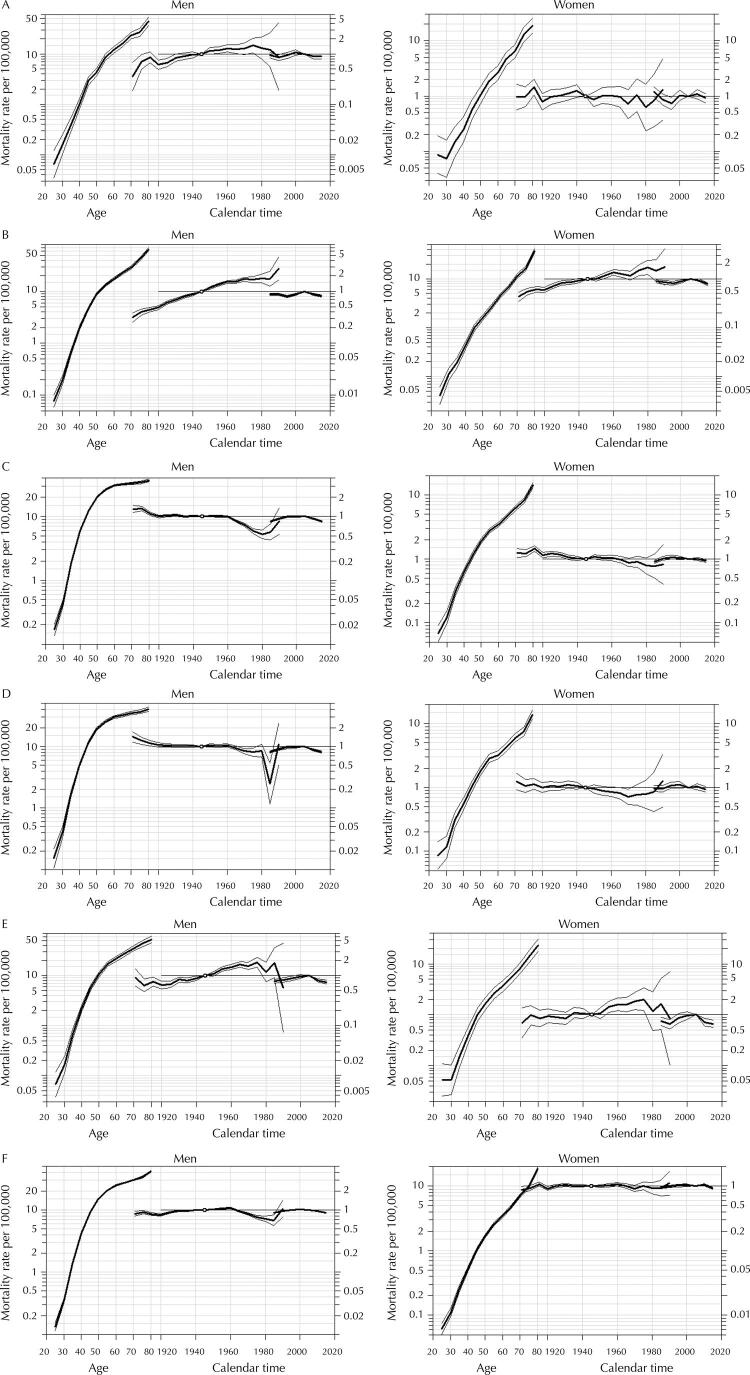
Adjusted effects and 95% confidence intervals of the APC model for mortality from oral and oropharyngeal cancer by sex and region, the first curve being the age effect, the second curve, the cohort effect, and the third, the period effect. North (A), Northeast (B), Southeast (C), South (D), Central-West (E), Brazil (F). Brazil, 1983–2017.

Regarding the cohort effect, the APC model ( Figure 3 ) shows an increased risk of mortality from oral and oropharyngeal cancer in the most recent cohorts and among men in the North, Northeast, and Central-West regions ( Figure 3A , 3B , and 3E ) compared to the reference cohort (born between 1943–1947). Mortality among women had the same effect but to a lesser extent. When analyzing the country as a whole ( Figure 3F ), the influence of age on mortality in older age groups can be observed, and an “average” cohort effect of the regions. There was no great magnitude in the effect of the period.

In the Southeast ( Figure 3C ) and South ( Figure 3D ) regions, the oldest cohorts had a higher risk of death. There is evidence of cohort effect on mortality from oral and oropharyngeal cancer. The younger cohorts present up to half of the reference cohort’s risk in the Southeast and up to a quarter of the reference cohort’s risk in the South.

## DISCUSSION

The present work is the first to analyze the APC effect on mortality from oral and oropharyngeal cancer in Brazil, covering the deaths registered since the establishment of the SIM. The analysis results show strong effects of age on mortality from oral and oropharyngeal cancer. The increase in mortality with age is noticeable even in younger age groups in the South and Southeast regions. There was an increased risk of mortality for the most recent cohorts in the North, Northeast, and Central-West regions. In the South and Southeast regions, these cohorts had a lower risk.

Mortality rates from oral and oropharyngeal cancer in men were five times higher than in women, similar to other studies on oral and oropharyngeal cancer mortality^15 , 16^ . The most raised hypothesis to explain the discrepancy between men and women is the exposure to the main risk factors. Tobacco consumption in Brazil has been higher among men than among women^17 , 18^ . Exposure to protective factors can also be considered, as men seek health services less frequently. Frequent consultation is an essential factor in timely diagnosing precancerous lesions and, consequently, preventing death from the disease^19^ .

The influence of age on the increased risk of mortality in all regions and both sexes is consistent with the fact that age is a known risk factor for several cancers and other NCDs^20^ . After 45 years of age, age effect in men stagnates, while this stagnation is not as abrupt in women. Still, mortality rates for men are higher than for women. The increase in mortality with age observed in younger age groups in the South and Southeast regions may be associated with exposure to more prevalent risk factors in this population. Genetic predisposition and HPV infection are important risk factors, as younger adults are generally less exposed to known carcinogens, such as tobacco and alcohol^21^ .

The marked differences between the North, Northeast, and Central-West regions with the Southeast and South regions show the cohort effect contributing to mortality from oral and oropharyngeal cancer when the younger cohorts are compared with the reference cohort. This effect may be linked to socioeconomic conditions since the more developed regions showed a decrease in the risk of death in the more recent cohorts, while in the less developed regions, the effect was the opposite. North and Northeast were characterized by the smaller number of healthcare professionals (physicians, nurses, and dentists) per 1 thousand inhabitants compared to the other regions, which present values twice as high for this indicator^22^ . This disparity impacts the population’s health status: in regions with greater difficulty accessing health services, screening, diagnosis, and timely treatment are complex, resulting in a worse prognosis and greater risk of death from cancer in general and by types of potentially curable cancers^23^ .

Investment and greater access to healthcare services in the South and Southeast may be a protective factor for younger cohorts, reducing mortality from oral and oropharyngeal cancer^24^ . Unlike what was observed in the North, Northeast, and Central-West regions, the effect observed in the South and Southeast regions is not linear. Until 1960, the cohort effect observed in Figures 3C , and 3D shows some stability. The risk reduction is concentrated in the later cohorts, representing the population aged around 40 years in the 1980s, when the prevalence of smoking began to decrease, and access to health services increased with the implementation of the Unified Health System.

Smoking, a significant risk factor associated with the incidence of oral and oropharyngeal cancer^21^ , has been decreasing since the 1980s. This decrease has been accompanied by a gradual migration of smoking from more favored populations to groups with lower socioeconomic status^25^ . However, changes in exposure to known risk factors are not the only explanation for the observed results. Although the prevalence of smoking in Brazil has decreased approximately 35% between 1989 and 2013^26^ , the risk of tobacco in mortality from oral and oropharyngeal cancer is not immediate. The cohorts that are currently being exposed may develop several outcomes in the coming years. Understanding the influence of risk factors on long-latency diseases requires reliable historical information about the distribution of these factors. While alcohol is also associated with oral cancer^21^ , few studies in Brazil analyze the population’s historical series of alcohol consumption and it was only from 1980 onwards that national information on tobacco consumption was available.

The progressive improvement in SIM may have also influenced the increased risk of mortality in the poorest regions. In the initial period of this system, coverage in the North and Northeast was low. In contrast, in the South and Southeast regions, on the contrary, coverage was higher from the beginning. Thus, death proportion with proper identification of the cause was already higher in these regions since the early 1980s^27^ . The present study corrected mortality by proportionally redistributing deaths from unspecified causes to partially correct this problem. It should also be noted that cancer is a long-latency pathology, with symptoms that require medical attention. Thus, access to healthcare services is essential for recognizing the disease and correctly filling out the underlying cause on the death certificate^12^ .

Concerning the effects observed in the APC analysis, the comparison with previous data was limited by the lack of studies in Brazil. Internationally, Bonifazi et al.^28^ observed that male mortality from oral cancer decreased in the European Union during 1970–2007. There was a decrease in the effects of cohorts born after the 1950s, reflecting changes in alcohol and tobacco consumption in various populations. However, Negri et al.^29^ observed a significant increase in the projections for mortality from oral and pharyngeal cancer in Europe from 2000 onwards.

Unlike Brazil, European countries have historical information on the distribution of alcohol and tobacco consumption, which allowed the authors of the articles mentioned above to conclude that the effects of the cohorts reflect the increases in the consumption of these substances in Eastern and Central Europe. In India, Shridhar et al.^30^ observed an upward trend in oral cancer mortality rates among men in Mumbai and period and cohort effects with higher effects among younger men.

It is essential to highlight some limitations of the present study. In Brazil, the quality of information on mortality varies between regions of the country. North and Northeast still have a large percentage of under-recorded or registered deaths with an ill-defined cause. The decrease in underreporting observed in recent years may affect the results obtained, giving the impression that the situation in these regions has worsened in more recent periods. SIM can also be affected by inadequate attribution of immediate causes or intermediate conditions as the underlying cause of death, usually referred to as “garbage codes”^31^ , a common limitation in studies based on secondary data.

However, underreporting’s influence was expected to be minimized by the proportional distribution of deaths from ill-defined causes in the analyzed data. It is also noteworthy that the quality and coverage of SIM data has gradually increased, which has been consolidating itself as a robust information system with broad national coverage. According to Datasus, significant efforts have been made, especially to reduce underreporting^12^ .

Despite its limitations, a study’s strength was the ability to analyze in isolation the effect of the cohort on mortality from oral and oropharyngeal cancer using the APC model, highlighting significant regional differences that should be considered in the development of public policies aimed at the population at risk. This is the first study to analyze the effects of age, period, and cohort on mortality from oral and oropharyngeal cancer in Brazil.

The analysis of cohort effects is particularly relevant concerning exposure to risk factors throughout life, making them a crucial element to explain rate behavior in chronic diseases^8^ . Previous studies using only trend analysis^15 , 16^ do not have the appropriate tools to verify the effect of birth cohorts on mortality. In the specific case of oral and oropharyngeal cancer, a disease with high rates in Brazil, with a highly preventable outcome, there is no other study in the literature analyzing the behavior of mortality from this disease for such an extended period, nor with the application of APC models. This was the first study to assess the trend in mortality from oral and oropharyngeal cancer in the last four decades.

Significantly reducing the risk of mortality in younger cohorts from less favored regions requires increasing access to healthcare services for timely diagnosis and treatment and a consequent reduction in deaths. This study shows the importance of implementing public policies to reduce oral and oropharyngeal cancer mortality that benefit the population at risk.
