# Supplementary material for: Mortality from oral and oropharyngeal cancer: age-period-cohort effect, Brazil, 1983–2017
Source: Rev Saude Publica. 2021 Oct 25;55:72. doi: 10.11606/s1518-8787.2021055003093 (PMC8555878; doi:10.11606/s1518-8787.2021055003093)
Supplement: Translation

# Translation: Mortalidade por câncer de boca e orofaringe: efeito idade-período-coorte, Brasil, 1983–2017

**Authors:** Lillia Magali Estrada Perea, José Leopoldo Ferreira Antunes, Marco Aurelio Peres

**Affiliations:** I Brasil Universidade Federal de Santa Catarina. Programa de Pós-Graduação em Saúde Coletiva. Florianópolis, SC, Brasil; II Brasil Universidade de São Paulo. Faculdade de Saúde Pública. São Paulo, SP, Brasil; III Singapore National Dental Research Institute Singapore. National Dental Centre Singapore. Singapore

**Keywords:** Neoplasias Orofaríngeas, Mortalidade, Efeito Idade, Efeito Período, Efeito de Coortes

## Abstract

**OBJETIVO:**

Estimar o efeito da idade, período e coorte de nascimento na mortalidade por câncer de boca e orofaringe no Brasil e suas macrorregiões.

**MÉTODO:**

Foram analisados os óbitos por câncer de boca e orofaringe de 1983 a 2017. Aplicou-se o modelo de regressão de Poisson, utilizando funções estimáveis propostas por Holford.

**RESULTADOS:**

No período de 1983 a 2017, foram registrados no Brasil 142.634 óbitos por câncer de boca e orofaringe, 81% entre o sexo masculino, e as regiões Sul e Sudeste apresentaram as taxas mais altas. Os maiores efeitos de período foram observados na mortalidade masculina das regiões Sudeste e Centro-Oeste para o período de referência 2003–2007. Nas regiões Norte, Nordeste e Centro-Oeste foi observado aumento do risco de mortalidade nas coortes masculinas mais recentes. Na região Norte o maior risco identificado foi para homens nascidos entre 1973 e 1977 (RR = 1,47; IC95% 1,05–2,08); no Nordeste, para homens nascidos entre 1988 e 1992 (RR = 2,77; IC95% 1,66–4,63); e no Centro-Oeste, para mulheres nascidas entre 1973 e 1977 (RR = 2,01; IC95% 1,19–3,39). Nas regiões Sudeste e Sul, as coortes mais recentes apresentaram taxas de mortalidade mais baixas. O menor risco na região Sudeste foi observado na coorte masculina nascida entre 1978 e 1982 (RR = 0,53; IC95% = 0,45–0,62), e entre 1983 e 1987 na região Sul (RR = 0,25; IC95% 0,12–0,54).

**CONCLUSÕES:**

A idade teve efeito significativo na mortalidade por câncer de boca e orofaringe em todas as regiões. Nas regiões Norte, Nordeste e Centro-Oeste, foi observado aumento do risco nas coortes mais recentes, enquanto nas regiões Sul e Sudeste essas coortes apresentaram risco menor quando comparadas às coortes mais antigas.

## INTRODUÇÃO

Como resultado do envelhecimento populacional que caracteriza a transição demográfica, aumentando a incidência de doenças crônicas não transmissíveis (DCNT), o perfil brasileiro de morbimortalidade tem mudado^1^ . Dentro do grupo das DCNT, em 2017 o câncer foi responsável por 56,9% das mortes no Brasil na faixa etária de 30 a 69 anos^2^ . O crescimento do gasto em saúde com as neoplasias observado nas últimas décadas decorre justamente da composição etária da população^1^ .

O câncer de boca e orofaringe é considerado um problema de saúde pública, especialmente no Brasil, país com as maiores taxas de mortalidade por esse tipo de câncer na América Latina^3^ . Aumento da incidência dessa neoplasia, assim como tendência de aumento de 1983 até 2002, tem sido observada predominantemente em países de alta renda^4^ .

Apesar do progresso em investigação e terapia, a sobrevida de entre 5 e 10 anos dos pacientes diagnosticados com câncer de boca e orofaringe não melhorou significativamente nos últimos anos^5^ . Em 2018, o câncer de boca teve a maior incidência entre todos os cânceres na Melanésia e no sul da Ásia entre os homens, e é a principal causa de mortalidade relacionada ao câncer entre homens na Índia e no Sri Lanka. Nos países de baixo Índice de Desenvolvimento Humano, o câncer de boca é o quarto tipo de câncer mais comum entre o sexo masculino^6 , 7^ .

A análise de indicadores através do tempo é útil na medida em que permite detectar fatores que afetam diferentemente os grupos populacionais. Para lidar com dados ou observações ordenadas ao longo do tempo, é necessário analisar e interpretar as contribuições de três fenômenos-chave: o efeito da idade; os efeitos de período; e os efeitos de diferenças no ano de nascimento, também chamados “efeitos de coorte”, por meio de estudo sistemático denominado “análise de idade-período-coorte” (APC, do inglês *age-period-cohort* )^8^ .

Estudos analisando o comportamento histórico da mortalidade por câncer de boca e faringe geralmente se limitam a analisar a série histórica das taxas padronizadas, indicadores úteis para mensurar o efeito da idade e do período. No entanto, tal análise deixa de lado o possível efeito da coorte de nascimento no comportamento das doenças crônicas.

O presente estudo tem como objetivo estimar pela primeira vez os efeitos da idade, período e coorte de nascimento na mortalidade por câncer de boca e orofaringe nas macrorregiões do Brasil.

## MÉTODOS

Trata-se de estudo ecológico da distribuição temporal da mortalidade por câncer de boca e orofaringe no Brasil e suas macrorregiões de 1983 a 2017, usando o modelo APC. O estudo considerou os óbitos de pessoas com 25 anos de idade ou mais, uma vez que casos de câncer de boca e orofaringe na população menor de 25 anos são raros (< 2% do total de casos). A primeira coorte analisada incluiu nascidos entre 1903 e 1907.

Os dados de mortalidade foram obtidos do Sistema de Informações sobre Mortalidade (SIM) do Datasus^9^ . Foram incluídos os óbitos por câncer de boca e orofaringe (140, 141, 143-146, 149 da CID 9ª Revisão, e C00-C06, C09, C10, C14 da CID 10ª Revisão) segundo a tabela de correspondências proposta por Fritz et al^10^ . Dados populacionais também foram obtidos no Datasus, com base nos censos de 1980, 1991, 2000 e 2010. As projeções para as populações dos anos intercensitários foram as estimadas pelo Instituto Brasileiro de Geografia e Estatística (IBGE)^11^ . Os dados do SIM são catalogados como dados secundários, sem identificação do nome dos pacientes e, portanto, não implicam risco para os participantes de pesquisa, suas informações ou seus familiares.

Estudos utilizando dados secundários podem ser afetados pela subnotificação no registro das informações. Para minimizar esse problema, os óbitos por causas mal definidas (códigos 780-799 da CID-9 e R00-R99 da CID-10) foram redistribuídos de modo proporcional aos casos de câncer de boca e orofaringe em cada ano, sexo e faixa etária^12^ . As faixas etárias utilizadas foram agrupadas em intervalos de 5 anos.

Para análise do modelo APC, os períodos foram agrupados em intervalos de cinco anos, totalizando sete períodos, e utilizou-se o modelo de regressão de Poisson. Esse modelo assume que o número esperado de óbitos segue uma distribuição de Poisson e pode ser expresso como uma regressão log-linear, como observado na equação [1]:

$$log\;(E_{ij})\;=\;log\;(P_{ij})\;+\;\mu \;+\;\alpha_{i\;+}\;\beta_{j\;+}\;{ϓ}_{k\;+}\;\varepsilon_{ij}$$ [1]

Onde ( *E*_ij_) denota o número esperado de óbitos no grupo ( *i* , *j* ), e o log de ( *P*_ij_) é a exposição ou tempo em que cada indivíduo esteve exposto ao risco, também chamada de *offset* ; *µ* representa o intercepto; α_i_representa o efeito do grupo idade *i* ; β_j_representa o efeito do período *j* ; e ϓ_k_o efeito da coorte *k* . O termo ε_ij_é relativo ao erro aleatório para a idade *i* e período *j* ^13^ .

A principal dificuldade para ajustar um modelo envolvendo idade, período e coorte é a relação linear entre eles, que configura um problema conhecido como “problema de não identificabilidade”. Não há consenso quanto à melhor forma de resolvê-lo. O presente estudo optou por estimar os parâmetros do efeito APC utilizando desvios, curvaturas e *drift* , método proposto por Holford^14^ , amplamente usado e aceito na literatura sobre mortalidade por câncer *.* Esse método sugere limitar a análise dos efeitos a suas combinações lineares e curvaturas. A tendência linear dos efeitos é dividida em um primeiro efeito linear associado a idade e um segundo efeito chamado *drift,* o efeito linear do período e da coorte.

A medida de associação gerada pelo modelo APC é o risco relativo (RR) de cada período, com referência ao período 2003–2007, e o RR de cada coorte, com referência à coorte de nascidos entre 1943 e 1947. Optou-se por essas referências considerando que as coortes e períodos centrais apresentam maior estabilidade^14^ . A estatística *deviance* foi utilizada para avaliar o ajuste do modelo. A contribuição dos efeitos foi avaliada pela comparação da *deviance* do modelo estimado com o efeito específico em relação ao modelo completo (idade-período-coorte). Os valores estatisticamente significativos foram determinados através de análise dos intervalos de confiança de 95%. As análises foram realizadas com a biblioteca Epi do *software* livre R (R Foundation for Statistical Computing, Viena, Áustria).

## RESULTADOS

No período de 1983 a 2017, foram registrados no Brasil 142.634 óbitos por câncer de boca e orofaringe, 139.924 (98,1%) entre pessoas com 25 anos ou mais. Dentre esses óbitos de indivíduos com idade superior a 25 anos, 81% ocorreram entre homens. As taxas de mortalidade para o sexo masculino foram, em média, cinco vezes maiores do que para o sexo feminino. A região Sudeste apresentou taxas duas vezes mais altas do que a região com as menores taxas, a região Nordeste.

As regiões Sudeste e Sul apresentaram as maiores taxas médias para o período, 6,4 por 100 mil habitantes e 6,2 por 100 mil habitantes homens, e 1,1 por 100 mil habitantes e 1 por 100 mil habitantes mulheres, respectivamente. As menores taxas médias para o período foram observadas na região Norte, 2,4 por 100 mil habitantes homens e 0,9 por 100 mil habitantes mulheres.

Como pode ser observado na Tabela 1 , o modelo APC apresentou o melhor ajuste quando comparado aos outros modelos (idade, idade- *drift* , idade-coorte, idade-período).

**Tabela 1:** Ajustes dos modelos do efeito APC para mortalidade por câncer de boca e orofaringe entre 1983 e 2017 no Brasil, segundo sexo e regiões.

Norte						

	Masculino			Feminino		

Modelo	Graus de liberdade	*Deviance*	p	Graus de liberdade	*Deviance*	p
Idade	72	136,56		72	109,97	
Idade- *drift*	71	95,43	< 0,001	71	109,97	0,994
Idade-coorte	55	74,57	0,184	55	90,27	0,234
Idade-período-coorte	**50**	**59,67**	**0,010**	**50**	**75,24**	**0,010**
Idade-período	66	81,29	0,156	66	97,10	0,147
Idade- *drift*	71	95,43	0,014	71	109,97	0,024

**Nordeste**						

	**Masculino**			**Feminino**		

**Modelo**	**Graus de liberdade**	***Deviance***	**p**	**Graus de liberdade**	***Deviance***	**p**

Idade	72	1408,09		72	359,20	
Idade- *drift*	71	265,50	< 0,001	71	180,35	< 0,001
Idade-coorte	55	171,60	< 0,001	55	125,04	< 0,001
Idade-período-coorte	**50**	**64,02**	**< 0,001**	**50**	**67,65**	**< 0,001**
Idade-período	66	118,25	< 0,001	66	97,01	0,021
Idade- *drift*	71	265,50	< 0,001	71	180,35	< 0,001

**Sudeste**						

	**Masculino**			**Feminino**		

**Modelo**	**Graus de liberdade**	***Deviance***	**p**	**Graus de liberdade**	***Deviance***	**p**

Idade	72	1105,14		72	204,61	
Idade- *drift*	71	847,40	< 0,001	71	135,36	< 0,001
Idade-coorte	55	380,05	< 0,001	55	77,68	< 0,001
Idade-período-coorte	**50**	**78,11**	**< 0,001**	**50**	**47,25**	**< 0,001**
Idade-período	66	397,09	< 0,001	66	91,98	< 0,001
Idade- *drift*	71	847,40	< 0,001	71	135,36	< 0,001

**Sul**						

	**Masculino**			**Feminino**		

**Modelo**	**Graus de liberdade**	***Deviance***	**p**	**Graus de liberdade**	***Deviance***	**p**

Idade	72	319,87		72	84,11	
Idade- *drift*	71	274,06	< 0,001	71	70,21	< 0,001
Idade-coorte	55	193,57	< 0,001	55	55,12	0,518
Idade-período-coorte	**50**	**64,10**	**< 0,001**	**50**	**42,33**	**0,025**
Idade-período	66	123,85	< 0,001	66	56,60	0,578
Idade- *drift*	71	274,06	< 0,001	71	70,21	0,018

**Centro-Oeste**						

	**Masculino**			**Feminino**		

**Modelo**	**Graus de liberdade**	***Deviance***	**p**	**Graus de liberdade**	***Deviance***	**p**

Idade	72	273,92		72	120,48	
Idade- *drift*	71	161,83	< 0,001	71	116,45	0,044
Idade-coorte	55	117,21	< 0,001	55	99,98	0,420
Idade-período-coorte	**50**	**55,05**	**< 0,001**	**50**	**66,85**	**< 0,001**
Idade-período	66	105,46	< 0,001	66	84,37	0,352
Idade- *drift*	71	161,83	< 0,001	71	116,45	< 0,001

Na Figura 1 é possível observar o comportamento das taxas de mortalidade dentro de cada faixa etária nos diferentes períodos analisados. É evidente o efeito da idade na mortalidade por câncer de boca e orofaringe em todas as regiões, uma vez que faixas etárias mais velhas apresentam sempre as taxas mais altas. O paralelismo observado entre as linhas das diferentes faixas etárias evidencia a ausência de forte efeito de período, o que também foi observado nos efeitos obtidos pelo modelo APC. Nas linhas inferiores, correspondentes às taxas de mortalidade para as faixas etárias mais jovens, é possível perceber a instabilidade das taxas devido ao baixo número de casos nesses grupos.

**Figura 1:** Taxas de mortalidade por câncer de boca e orofaringe por período, conectadas dentro de cada faixa etária, segundo sexo e região. Brasil, 1983–2017.

Já a Figura 2 mostra as taxas de mortalidade dentro de cada faixa etária, mas dessa vez segundo as coortes analisadas. Nas regiões Norte ( Figura 2A ), Nordeste ( Figura 2B ) e Centro-Oeste ( Figura 2E ) é possível observar inclinação positiva das linhas correspondentes a cada faixa etária, o que indica aumento das taxas para as coortes mais novas. De forma inversa, nas regiões Sudeste ( Figura 2C ) e Sul ( Figura 2D ) essa inclinação é negativa. Esse resultado, no que tange às coortes, também foi encontrado nos efeitos obtidos pelo modelo APC. Mais uma vez, as linhas inferiores mostram instabilidade das taxas devido ao pouco número de casos nesses grupos.

**Figura 2:** Taxas de mortalidade por câncer de boca e orofaringe por coorte conectadas dentro de cada faixa etária, segundo sexo e região. Brasil, 1983–2017.

Nos resultados obtidos pelo modelo APC foi possível observar que a idade é o efeito que mais influencia as taxas de mortalidade por câncer de boca e orofaringe. Nas regiões Norte, Nordeste e Centro-Oeste foi observado aumento significativo da mortalidade a partir de idades mais avançadas (45 anos para homens e 60 para mulheres). O aumento da mortalidade não se comporta de maneira linear em todas as faixas etárias, e a inclinação da reta muda nas faixas etárias mais velhas. Nas regiões Sudeste e Sul, o aumento de risco de morte já é presente em faixas etárias mais jovens, a partir dos 35 anos no sexo masculino e dos 45 no sexo feminino. Quando analisado o país como um todo, observa-se aumento significativo da mortalidade a partir dos 40 anos de idade para o sexo masculino e 50 anos para o sexo feminino.

Não foram observados efeitos de período significativos para as regiões Norte e Sudeste. No Nordeste, todos os períodos apresentaram menor risco quando comparados com a referência, e o período de menor risco foi 1993–1997 para homens (RR = 0,80, IC95% 0,76–0,84) e 2013–2017 para mulheres (RR = 0,78, IC95% 0,72–0,83). No Sul, o efeito-período só foi observado entre homens, que durante os períodos 1983–1987 e 1988–1993 apresentaram menor risco em comparação com a referência (RR = 0,81, IC95% 0,78–0,85; e RR = 0,89, IC95% 0,86–0,94, respectivamente). No Centro-Oeste, o período de maior risco foi o período-referência.

No que tange ao efeito de coorte, o modelo APC ( Figura 3 ) mostra aumento do risco de mortalidade por câncer de boca e orofaringe nas coortes mais recentes e entre o sexo masculino nas regiões Norte, Nordeste e Centro-Oeste ( Figura 3A , 3B e 3E ) em comparação com a coorte-referência (nascidos entre 1943–1947). A mortalidade entre as mulheres teve o mesmo efeito, mas com menor magnitude. Quando analisado o país como um todo ( Figura 3F ), pode se observar a influência da idade na mortalidade nas faixas etárias mais velhas, assim como um efeito de coorte “médio” das regiões. Não foi observada grande magnitude no efeito do período.

**Figura 3:** Efeitos ajustados e intervalos de confiança de 95% do modelo APC para a mortalidade por câncer de boca e orofaringe segundo sexo e região, sendo a primeira curva o efeito da idade, a segunda curva o efeito da coorte e a terceira curva o efeito do período. Norte (A), Nordeste (B), Sudeste (C), Sul (D), Centro-Oeste (E), Brasil (F). Brasil, 1983–2017.

Nas regiões Sudeste ( Figura 3C ) e Sul ( Figura 3D ), as coortes mais antigas apresentaram maior risco de óbito. Ou seja, há evidência do efeito de coorte na mortalidade por câncer de boca e orofaringe, uma vez que as coortes mais novas chegam a apresentar a metade do risco da coorte-referência no Sudeste e até um quarto do risco da coorte-referência no Sul.

## DISCUSSÃO

O presente trabalho é o primeiro a analisar o efeito APC na mortalidade por câncer de boca e orofaringe no Brasil abrangendo os óbitos registrados desde a conformação do SIM. Os resultados da análise mostram fortes efeitos da idade na mortalidade por câncer de boca e orofaringe, e o aumento da mortalidade com a idade é perceptível mesmo em faixas etárias mais jovens nas regiões Sul e Sudeste. Houve aumento do risco de mortalidade para as coortes mais recentes nas regiões Norte, Nordeste e Centro-Oeste. Nas regiões Sul e Sudeste, essas coortes apresentaram risco menor.

As taxas de mortalidade por câncer de boca e orofaringe nos homens foram cinco vezes maiores do que nas mulheres, resultado similar aos de outros estudos que analisaram a mortalidade por câncer de boca e orofaringe^15 , 16^ . A hipótese mais levantada para explicar a discrepância entre homens e mulheres é a exposição aos principais fatores de risco. O consumo de tabaco no Brasil tem sido maior entre homens do que entre mulheres^17 , 18^ . A exposição a fatores protetores também pode ser considerada, uma vez que homens procuram com menor frequência os serviços de saúde. A consulta frequente tem se mostrado um fator importante para diagnosticar oportunamente lesões pré-cancerosas e, consequentemente, evitar o óbito pela doença^19^ .

A influência da idade no aumento do risco de mortalidade em todas as regiões e em ambos os sexos condiz com o fato de a idade ser fator de risco conhecido para diversos cânceres e outras DCNT^20^ . Após os 45 anos, o efeito da idade no sexo masculino parece estagnar, enquanto nas mulheres essa estagnação não é tão abrupta. Ainda assim, as taxas de mortalidade no sexo masculino são maiores do que no sexo feminino. O aumento da mortalidade com a idade observado em faixas etárias mais jovens nas regiões Sul e Sudeste pode estar associado à exposição a fatores de risco mais prevalentes nessa população. A predisposição genética e a infecção por HPV têm se mostrado fatores de risco importantes, uma vez que adultos mais jovens em geral estão menos expostos a substâncias cancerígenas conhecidas, como tabaco e álcool^21^ .

As marcantes diferenças das regiões Norte, Nordeste e Centro-Oeste com as regiões Sudeste e Sul mostram que, de fato, há contribuição do efeito de coorte na mortalidade por câncer de boca e orofaringe quando as coortes mais novas são comparadas com a coorte-referência. Esse efeito pode estar ligado a condições socioeconômicas, uma vez que as regiões mais desenvolvidas apresentaram diminuição do risco de óbito nas coortes mais recentes, enquanto nas regiões menos desenvolvidas o efeito foi oposto. Norte e Nordeste se caracterizaram pelo menor número de profissionais de saúde (médicos, enfermeiros e odontólogos) por 1 mil habitantes em comparação com as demais regiões, que apresentam valores duas vezes maiores para esse indicador^22^ . Essa disparidade tem consequências no estado de saúde da população: nas regiões com maior dificuldade de acesso a serviços de saúde, o rastreio, o diagnóstico e o tratamento oportuno são dificultados, acarretando pior prognóstico e maior risco de óbito por câncer em geral e por tipos de câncer potencialmente curáveis^23^ .

O investimento e o maior acesso a serviços de saúde no Sul e no Sudeste podem ser fator protetor para as coortes mais novas, diminuindo a mortalidade por câncer de boca e orofaringe^24^ . Diferentemente do observado nas regiões Norte, Nordeste e Centro-Oeste, o efeito observado nas regiões Sul e Sudeste não é linear. Até 1960, o efeito-coorte observado nas Figuras 3C e 3D apresenta certa estabilidade. A diminuição do risco se concentra nas coortes posteriores, que representam a população com idade ao redor de 40 anos na década de 1980, quando a prevalência de tabagismo começa a diminuir e o acesso a serviços sanitários aumenta com a implantação do Sistema Único de Saúde.

O tabagismo, importante fator de risco associado à incidência de câncer de boca e orofaringe^21^ , vem diminuindo desde a década de 1980. Essa diminuição tem sido acompanhada por uma migração gradual do tabagismo das populações mais favorecidas para grupos com menor nível socioeconômico^25^ . No entanto, mudanças na exposição aos fatores de risco conhecidos não são a única explicação para os resultados observados. Embora a prevalência de tabagismo no Brasil tenha mostrado diminuição de aproximadamente 35% entre 1989 e 2013^26^ , o risco do tabaco na mortalidade por câncer de boca e orofaringe não é imediato. As coortes que estão sendo expostas atualmente poderão vir a desenvolver diversos desfechos nos próximos anos. Para entender a influência dos fatores de risco em doenças de longa latência, é necessário informação histórica confiável sobre a distribuição desses fatores. O álcool também é associado ao câncer bucal^21^ , mas no Brasil existem poucos estudos que analisem a série histórica do consumo de álcool pela população, e só a partir de 1980 é que se tem informação nacional sobre o consumo de tabaco.

O risco aumentado de mortalidade nas regiões mais pobres também pode ter sido influenciado pela progressiva melhora do SIM. No período inicial desse sistema, a cobertura no Norte e no Nordeste era baixa, enquanto nas regiões Sul e Sudeste, pelo contrário, a cobertura era mais elevada desde o início, de modo que a proporção de óbitos com devida identificação da causa já era maior nessas regiões desde o início dos anos 1980^27^ . Com a finalidade de corrigir parcialmente esse problema, o presente trabalho corrigiu a mortalidade pela redistribuição proporcional dos óbitos por causas não especificadas. Salienta-se também que o câncer é uma patologia de longa latência, com sintomas que requerem atenção médica. Assim, o acesso a serviços de saúde é importante para o reconhecimento da doença e correto preenchimento de causa básica na declaração de óbito^12^ .

Quanto aos efeitos observados na análise APC, a comparação com dados prévios se viu limitada pela falta de estudos no Brasil. No âmbito internacional, Bonifazi et al.^29^ observaram que a mortalidade masculina por câncer de boca diminuiu na União Europeia durante o período 1970–2007. Houve queda nos efeitos das coortes nascidas após a década de 1950, refletindo as mudanças no consumo de álcool e tabaco em várias populações. Entretanto, Negri et al.^28^ observaram grande aumento nas projeções para mortalidade por câncer de boca e faringe na Europa a partir de 2000.

Diferentemente do Brasil, os países europeus contam com informação histórica sobre a distribuição de consumo de álcool e tabaco, o que permitiu aos autores dos artigos mencionados concluir que os efeitos das coortes refletem os aumentos no consumo dessas substâncias na Europa Oriental e Central. Na Índia, Shridhar et al.^30^ observaram tendência de aumento nas taxas de mortalidade por câncer de boca nos homens de Mumbai, assim como efeitos de período e coorte com efeitos mais altos entre homens mais jovens.

É importante destacar algumas limitações do presente estudo. No Brasil, a qualidade da informação sobre mortalidade varia entre as regiões do país. Norte e Nordeste ainda têm grande percentual de óbitos sub-registrados ou registrados com causa mal definida. A diminuição da subnotificação observada nos últimos anos pode afetar os resultados obtidos, dando impressão de agravamento da situação nessas regiões em períodos mais recentes. O SIM também pode ser afetado pela atribuição inadequada de causas imediatas ou condições intermediárias como causa básica do óbito, o que é usualmente referido como “códigos-lixo” ( *garbage codes* )^31^ , uma limitação comum em estudos baseados em dados secundários.

No entanto, espera-se que a influência da subnotificação tenha sido minimizada pela distribuição proporcional de óbitos por causa mal definida nos dados analisados. Destaca-se também que têm aumentado gradativamente a qualidade e a abrangência dos dados do SIM, que vem se consolidando como sistema de informação forte e de ampla cobertura nacional. Segundo o Datasus, grandes esforços têm sido feitos especialmente para diminuir a subnotificação^12^ .

Apesar de suas limitações, o estudo teve como ponto forte a capacidade de analisar isoladamente o efeito da coorte na mortalidade por câncer de boca e orofaringe por meio do modelo APC, evidenciando grandes diferenças regionais que devem ser consideradas na elaboração de políticas públicas direcionadas à população em risco. Trata-se do primeiro estudo de análise dos efeitos de idade, período e coorte para a mortalidade por câncer de boca e orofaringe no Brasil.

A análise dos efeitos de coorte são particularmente relevantes no que tange à exposição a fatores de risco ao longo da vida, o que faz deles um elemento de grande importância para explicar o comportamento das taxas nas doenças crônicas^8^ . Estudos prévios que utilizam apenas análise de tendência^15 , 16^ não têm as ferramentas adequadas para verificar o efeito das coortes de nascimento na mortalidade. No caso específico de câncer de boca e orofaringe, uma doença com altas taxas no Brasil, com desfecho altamente prevenível, não há na literatura outro estudo analisando o comportamento da mortalidade por essa doença por um período tão longo, nem com aplicação de modelos APC. Este foi o primeiro estudo a avaliar a tendência de mortalidade por câncer de boca e orofaringe nas últimas quatro décadas.

Para diminuir significativamente o risco de mortalidade nas coortes mais novas das regiões menos favorecidas é necessário aumentar o acesso a serviços de saúde para diagnóstico e tratamento oportuno e consequente redução do óbito. O presente estudo mostra a importância de implantar políticas públicas para redução da mortalidade por câncer de boca e orofaringe que realmente beneficiem a população em risco.
